# Integrated Transcriptomic, In Silico, and In Vitro Characterization of lncRNA ENST00000615487.1 Reveals Epithelial-Specific Expression, Differential Subcellular Distribution Between Normal and Colorectal Cancer Cells, and Potential Regulatory Functions

**DOI:** 10.3390/ncrna12040024

**Published:** 2026-07-17

**Authors:** Nataša Đokić, Anastasija Bubanja, Jelena Karanović, Jovana Despotović

**Affiliations:** Gene Regulation in Cancer Group, Institute of Molecular Genetics and Genetic Engineering, University of Belgrade, 11000 Belgrade, Serbia; djokicnatasa13@gmail.com (N.Đ.); anastasija.bubanja@imgge.bg.ac.rs (A.B.); jelena.karanovic@imgge.bg.ac.rs (J.K.)

**Keywords:** ENST00000615487.1, long non-coding RNA, colorectal cancer, subcellular localization, bioinformatic analysis

## Abstract

**Background/Objectives**: Long non-coding RNAs (lncRNAs) are important regulators of tumor biology through their interactions with DNA, proteins, and non-coding RNAs. Although ENST00000615487.1 (also known as CTD-2396E7.11/AC010503.4) has been associated with multiple malignancies, its biological role in colorectal cancer (CRC) remains poorly characterized. This study aimed to investigate the expression pattern, cellular and subcellular localization, and potential functional role of ENST00000615487.1 in CRC using integrated in vitro and in silico approaches. **Methods**: Molecular characteristics of the transcript were obtained with the CPC2 and RNA Analyzer 3 tools. Differential expression of ENST00000615487.1 across 10 tumor types was analyzed using the UCSC Xena Browser. Transcript expression was experimentally evaluated in normal, tumor, and fibroblastic colon cell lines by PCR, while subcellular localization was assessed through the lncATLAS, lncLocator, and iLoc-LncRNA tools, and experimentally confirmed by qRT-PCR. Single-cell RNA sequencing data from the GSE161277 dataset were analyzed to determine cell type-specific expression patterns. Potential interactions with DNA, miRNAs, and proteins were investigated using Fasim-LongTarget, miRDB, and AnnoLnc2, followed by functional enrichment analyses using STRING and Enrichr. **Results**: ENST00000615487.1 was identified as a structurally stable non-coding transcript with a highly organized secondary structure. Differential expression analysis demonstrated significant downregulation in CRC compared with that in normal colon tissue. Single-cell transcriptomic analysis revealed predominantly epithelial-specific expression. In silico and experimental analyses demonstrated predominant nuclear localization in normal colon cells, whereas cytoplasmic enrichment was observed in CRC cells. Functional analyses identified potential interactions with *HIP1R*, *RPH3AL*, specific miRNAs, and proteins involved in transcriptional regulation and RNA processing pathways, as well as functional connections with proteins involved in vesicular transport. **Conclusions**: ENST00000615487.1 is a structurally stable lncRNA exhibiting context-dependent expression and localization patterns in CRC, suggesting a potential shift from nuclear transcriptional regulation toward cytoplasmic post-transcriptional functions during colorectal carcinogenesis.

## 1. Introduction

Colorectal cancer (CRC) is the third most commonly diagnosed malignancy in humans (9.6%), while ranking second in terms of mortality (9.3%) worldwide. In 2022, more than 1.9 million new CRC cases and approximately 904,000 deaths were reported worldwide. This makes CRC responsible for 1 in 10 cancer-related deaths [1]. Despite significant advances in surgical treatment, chemotherapy, targeted therapies, and immunotherapy, CRC remains associated with high morbidity and mortality, particularly in advanced stages of the disease. Early detection significantly improves patient survival; however, currently available diagnostic and prognostic approaches still show important limitations regarding sensitivity, specificity, and prediction of disease progression. Therefore, contemporary CRC research is increasingly focused on identifying novel molecular biomarkers and regulatory mechanisms involved in colorectal tumorigenesis, with the aim of improving diagnostics, prognostic assessment, and therapeutic strategies. Among these, long non-coding RNAs (lncRNAs) have emerged as important regulators of cancer-related biological processes, given the diversity of biological mechanisms in which they participate [2].

lncRNAs are a diverse class of non-protein-coding transcripts. Although they have historically been defined as transcripts longer than 200 nucleotides, recent consensus recommendations emphasize that this threshold is largely operational and suggest the use of more stringent criteria, including transcript lengths exceeding 500 nucleotides [3]. Despite the fact that they do not code for proteins, they represent an important link in numerous cellular processes [4]. They participate in the regulation of gene expression through epigenetic modifications and interactions with microRNA (miRNA) molecules and proteins [5], playing crucial roles in transcriptional and post-transcriptional processes. The deregulation of lncRNA molecules is intertwined with core processes of CRC development such as proliferation, apoptosis, metastasis, invasion, and angiogenesis [6]. A growing body of evidence indicates that lncRNAs play a key role in CRC tumorigenesis and progression. Bioinformatic analyses of RNA sequencing (RNA-seq) data, such as those from the TCGA database, have identified hundreds of differentially expressed lncRNAs in colorectal tumor tissue compared to healthy samples [7]. Since aberrant lncRNA expression in tissue and circulation reflects the disease stage and progression, these molecules emerge as key factors in advancing diagnostics and prognosis [5].

According to the Ensembl database, the transcript ENST00000615487.1 is classified as an lncRNA. It is the sole product of the gene ENSG00000275234.1, located on the forward strand of chromosome 19. It is listed as a novel transcript according to the Ensembl database, and represents an antisense transcript relative to the *DENND1C* gene. In the literature and available databases, it appears under various aliases, namely ENST00000615487.1 (Ensembl genome browser), CTD-2396E7.11 [8,9,10,11,12] and AC010503.4 [13,14,15,16,17,18]. The transcript ENST00000615487.1 has been identified as a significant regulatory molecule in different malignancies, including renal, ovarian, breast, endometrial, bladder, and gastric cancers, as well as melanoma [8,9,10,11,12,13,14,15,16,17,18]. In these systems, it participates in key processes such as metabolic reprogramming [8], ferroptosis [9], and autophagy [13], predominantly exerting a protective role and serving as a favorable prognostic factor associated with better disease outcomes and lower tumor grades [13,14,15]. In CRC, this transcript has been defined as one of the four key “hub” lncRNAs associated with disease progression. Its expression is highest in normal tissue, while progressively decreasing during the transition from adenoma to carcinoma. Elevated expression in normal tissues indicates a protective role of the transcript in the onset, development, and prognosis of CRC [11]. In contrast to the results of studies conducted on CRC tissue, our previous Next-Generation Sequencing (NGS) results obtained from HCEC-1CT, HCT116, SW620, and DLD1 cell lines grown as spheroids indicate an elevated expression of the ENST00000615487.1 transcript in CRC cell lines [7]. These contradictory results, along with the insufficient investigation of the function of the ENST00000615487.1 transcript, served as the main motivation for conducting this research. Considering previous evidence suggesting a potentially important role of ENST00000615487.1 in tumorigenesis, alongside conflicting findings regarding its expression in normal and tumor tissues, this study aimed to characterize the expression profile, cellular and subcellular localization, and to generate hypotheses regarding the potential regulatory functions of ENST00000615487.1 across different malignancies, with particular focus on CRC, through integrated in vitro and in silico analyses.

## 2. Results

### 2.1. Characteristics of ENST00000615487.1 Transcript

The structural characteristics of ENST00000615487.1 were analyzed using the Coding Potential Calculator 2 (CPC2) and RNA Analyzer 3 tools. Analyses included the assessment of coding potential, prediction of secondary structure stability, and structural features such as loop and hairpin formation. The results indicated that ENST00000615487.1 is a highly structured non-coding RNA transcript consisting of a single exon and spanning 688 base pairs (bp), consistent with its annotation as a lncRNA. The obtained results are summarized in Table 1.

### 2.2. Expression of ENST00000615487.1

#### 2.2.1. In Silico Analysis of Differential Expression of ENST00000615487.1 in Tumor Tissues

**Figure 1:**
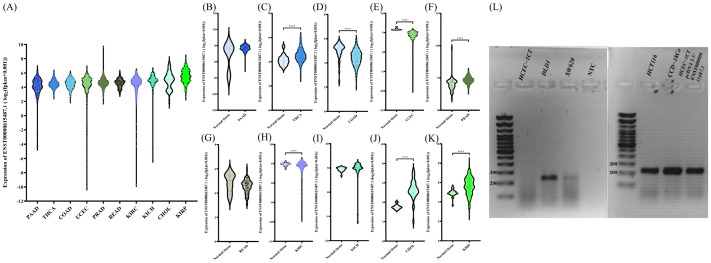
Expression analysis of ENST00000615487.1 across human malignancies and colon-derived cell lines. (**A**) Expression levels of ENST00000615487.1 across the 10 tumor types exhibiting the highest transcript expression, arranged in ascending order. Data were obtained using the UCSC Xena browser. (**B**–**K**) Differential expression analysis of ENST00000615487.1 between normal and tumor tissues in the selected tumor types: (**B**) PAAD, (**C**) THCA, (**D**) COAD, (**E**) PRAD, (**F**) UCEC, (**G**) READ, (**H**) KIRC, (**I**) KICH, (**J**) CHOL, and (**K**) KIRP. Data were obtained using the UCSC Xena browser. **** designates *q* < 0.0001; q-values represent FDR-adjusted *p*-values (Benjamini–Krieger–Yekutieli correction). (**L**) Detection of ENST00000615487.1 expression in normal, tumor, and fibroblastic colon cell lines by PCR. Agarose gel electrophoresis showing PCR products obtained from HCEC-1CT, HCT116, DLD1, SW620, and CCD-18Co cell lines. The first lane represents the 100 bp molecular weight marker. HCEC-1CT cells transfected with the pcDNA3.1/ENST00000615487.1 construct were used as a positive control, while NTC represents the negative control.

Differential expression analysis of ENST00000615487.1 was performed using the UCSC Xena browser. Based on transcript expression levels, the 10 tumor types exhibiting the highest expression of ENST00000615487.1 were selected for further analysis (Figure 1A), while tumor names, their abbreviations together with the number of samples of each tumor, and paired normal tissues are listed in Table S1. Differential expression between normal and tumor tissues within these tumor groups was subsequently examined (Figure 1B–K). A statistically significant upregulation of ENST00000615487.1 expression in tumor tissue was observed in THCA, CHOL, PRAD, and KIRP (all FDR-adjusted *q* < 0.001). In contrast, significantly reduced expression in tumor tissue was detected in COAD, UCEC, and KIRC (all FDR-adjusted *q* < 0.001). No statistically significant differences in ENST00000615487.1 expression between normal and tumor tissues were observed in PAAD (FDR-adjusted *q* = 0.136), READ (FDR-adjusted *q* = 0.110), and KICH (FDR-adjusted *q* = 0.244).

#### 2.2.2. Detection of ENST00000615487.1 in Normal, Tumor, and Fibroblastic Colon Cells

The expression of ENST00000615487.1 was assessed in HCEC-1CT, HCT116, DLD1, SW620, and CCD-18Co cell lines by PCR using cDNA synthesized from total RNA. Expression of ENST00000615487.1 was detected in the fibroblastic cell line CCD-18Co and in all examined CRC cell lines, whereas no expression was detected in the normal colon epithelial cell line HCEC-1CT (Figure 1L).

#### 2.2.3. Single-Cell Transcriptomic Analysis of ENST00000615487.1 in Colorectal Cancer

**Figure 2:**
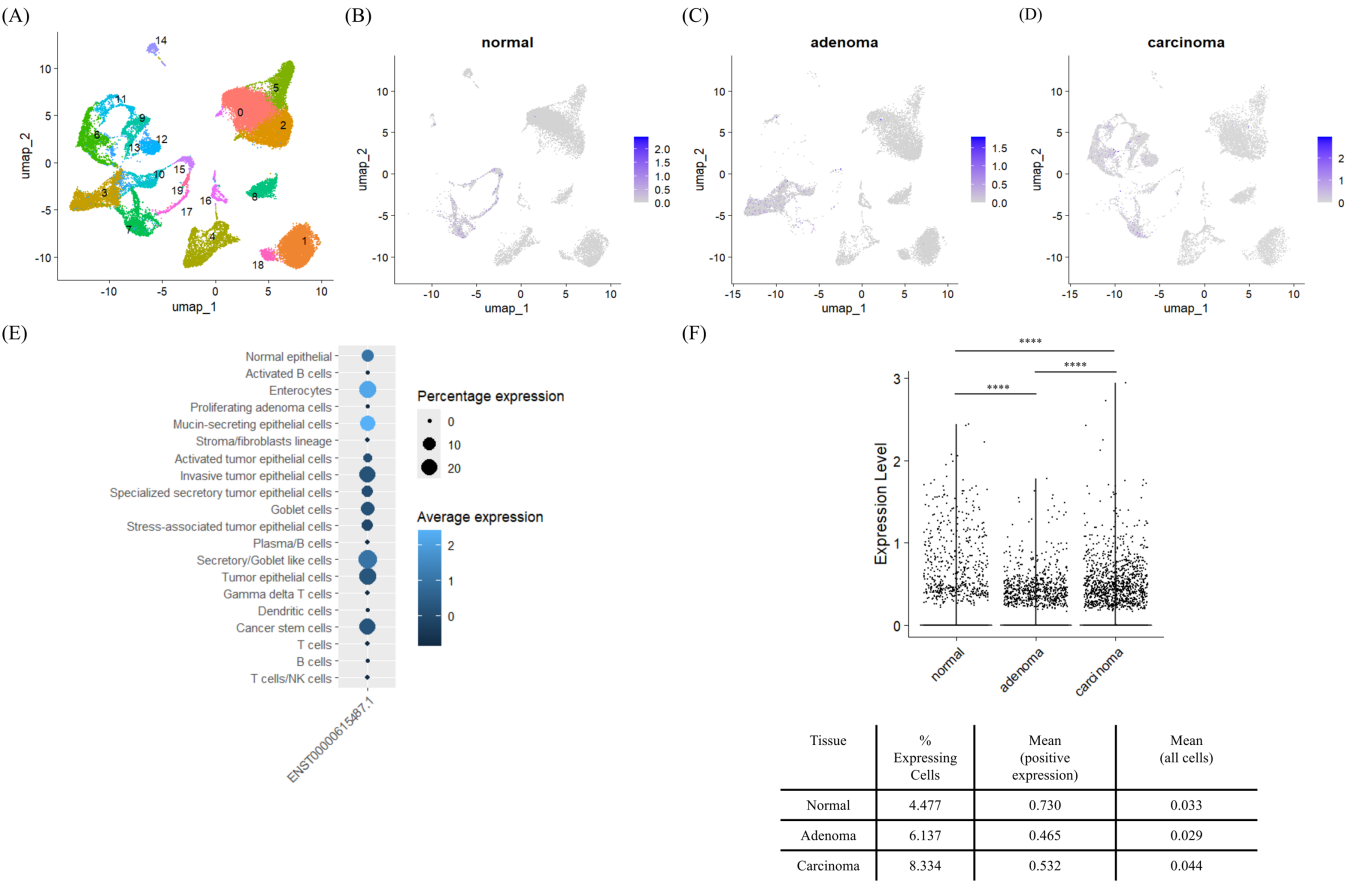
Single-cell expression landscape of ENST00000615487.1 in CRC. (**A**) UMAP visualization of the annotated single-cell populations identified in carcinoma, adenoma, blood, and adjacent normal tissue samples. Cluster identities are indicated by numbers corresponding to the following cell populations: 0—T cells/NK cells; 1—B cells; 2—T cells; 3—cancer stem cells; 4—dendritic cells; 5—gamma delta T cells; 6—tumor epithelial cells; 7—secretory/goblet-like cells; 8—plasma/B cells; 9—stress-associated tumor epithelial cells; 10—goblet cells; 11—specialized secretory tumor epithelial cells; 12—invasive tumor epithelial cells; 13—activated tumor epithelial cells; 14—stromal/fibroblast lineage cells; 15—mucin-secreting epithelial cells; 16—proliferating adenoma cells; 17—enterocytes; 18—activated B cells; and 19—normal epithelial cells. (**B**–**E**) UMAP feature plots illustrating ENST00000615487.1 expression across cell clusters in normal (**B**), adenoma (**C**), and carcinoma (**D**). (**E**) Dot plot showing ENST00000615487.1 expression across annotated cell populations. Dot size represents the percentage of expressing cells, while color intensity indicates average expression levels. (**F**) Comparative analysis of ENST00000615487.1 expression across normal, adenoma and carcinoma tissue conditions demonstrating differences in both the proportion of expressing cells and mean transcript expression in normal, adenoma, and carcinoma samples. **** designates *p* < 0.0001.

Single-cell RNA sequencing analysis of normal (11,817 cells), adenoma (11,618 cells), carcinoma (12,395 cells), and blood-derived (2134) samples identified several distinct cellular populations. UMAP visualization revealed distinct immune, stromal and epithelial populations, including T cells, B cells, dendritic cells, fibroblast/stromal cells, goblet cells, enterocytes and several tumor-associated epithelial states (Figure 2A). Several transcriptionally distinct tumor epithelial populations were identified, including stem-like, stress-associated, invasive, secretory-like and activated tumor epithelial states (Figure 2E). Analysis of the proportion of expressing cells revealed that ENST00000615487.1 was detected in 4.48% of normal cells, 6.14% of adenoma cells, and 8.33% of carcinoma cells. DotPlot analysis demonstrated that ENST00000615487.1 was expressed in a relatively small subset of cells and was predominantly restricted to epithelial populations, particularly enterocyte-like, goblet-like, and mucin-secreting epithelial states, while showing minimal expression in immune and stromal compartments. Expression of ENST00000615487.1 was also detected in invasive, stress-associated, and activated tumor epithelial populations (Figure 2E). The ENST00000615487.1 transcript exhibited low overall expression across all tissue groups (Figure 2B–D). However, when analysis was restricted to expressing cells only, mean expression levels increased substantially. Comparative analysis demonstrated distinct expression dynamics across tissue conditions, with carcinoma samples showing a higher proportion of expressing cells, whereas transcript-positive normal cells exhibited higher mean expression intensity relative to adenoma and carcinoma populations (*p* < 0.001), suggesting altered regulation of ENST00000615487.1 during CRC development (Figure 2F).

### 2.3. Determination of ENST00000615487.1 Subcellular Localization

#### 2.3.1. In Silico Prediction of ENST00000615487.1 Subcellular Localization

**Figure 3:**
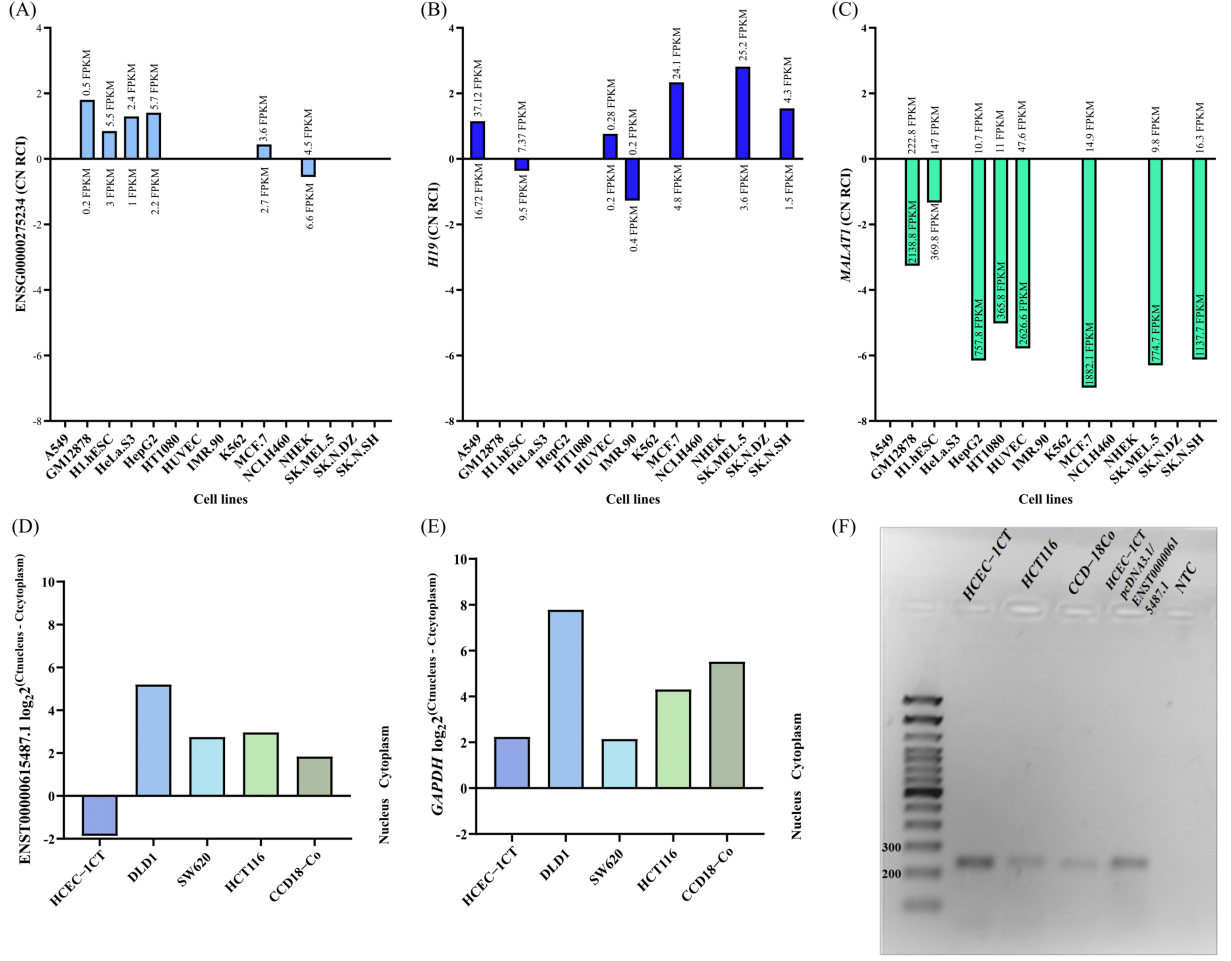
Subcellular and extracellular localization of ENST00000615487.1. (**A**–**C**) In silico prediction of ENST00000615487.1 subcellular localization (**A**) based on lncATLAS cytoplasm-to-nucleus relative concentration index (CN RCI) across multiple cell lines. Positive CN RCI values indicate predominant cytoplasmic localization, while negative values indicate nuclear enrichment. H19 (**B**) and MALAT1 (**C**) were included as cytoplasmic and nuclear reference transcripts, respectively, to validate dataset reliability. (**D**,**E**) Experimental validation of ENST00000615487.1 subcellular localization (**D**) by qRT-PCR in nuclear and cytoplasmic fractions of normal colon epithelial (HCEC-1CT), CRC (HCT116, DLD1, SW620), and fibroblastic (CCD-18Co) cell lines. (**E**) *GAPDH* served as a cytoplasmic fractionation control. (**F**) Detection of ENST00000615487.1 in exosomal RNA isolated from HCEC-1CT, HCT116, and CCD-18Co cell lines, confirming its presence in extracellular vesicles. The first lane represents the 100 bp molecular weight marker. HCEC-1CT pcDNA3.1/ENST00000615487.1 was used as a positive control, while NTC served as the negative control.

Analysis using lncATLAS indicated predominant cytoplasmic localization of ENST00000615487.1, as reflected by positive cytoplasm-to-nucleus relative concentration index (CN RCI) values in most analyzed cell lines (CN RCI > 0) (Figure 3A). The only exception was the Normal Human Epidermal Keratinocyte (NHEK) cell line, in which a negative CN RCI value (CN RCI < 0) suggested predominant nuclear localization. To assess the reliability of the analyzed cellular subfractions, reference transcripts with known localization patterns were included. *H19*, used as a cytoplasmic marker, showed predominant cytoplasmic localization in most analyzed cell lines (Figure 3B), whereas *MALAT1*, used as a nuclear marker, consistently exhibited nuclear localization across all cell lines (Figure 3C). Consistent with the lncATLAS results, analysis using lncLocator predicted the cytoplasm (probability score of 0.335) as the most probable subcellular compartment for ENST00000615487.1 localization. Additionally, iLoc-LncRNA (2.0) predicted potential ribosome-associated localization of the transcript (probability score of 0.862).

#### 2.3.2. Experimental Validation of ENST00000615487.1 Localization in Subcellular and Extracellular Fractions of Normal, Tumor, and Fibroblastic Colon Cells

The presence of ENST00000615487.1 in nuclear and cytoplasmic fractions, as well as in exosomes derived from HCEC-1CT, HCT116, DLD1, SW620, and CCD-18Co cell lines, was analyzed by qRT-PCR. *GAPDH* expression was used as a control to validate successful subcellular fractionation. In CRC cell lines and in the fibroblastic cell line CCD-18Co, ENST00000615487.1 expression was predominantly detected in the cytoplasmic fraction compared to the nuclear fraction. In contrast, the normal colon epithelial cell line HCEC-1CT exhibited predominant nuclear localization of ENST00000615487.1 (Figure 3D). As expected, *GAPDH* expression was enriched in cytoplasmic fractions across all analyzed cell lines (Figure 3E).

Semi-quantitative PCR analysis of exosomal RNA isolated from the media of HCEC-1CT, HCT116, and CCD-18Co cell lines confirmed the presence of ENST00000615487.1 in extracellular vesicles derived from all three cell lines (Figure 3F).

### 2.4. In Silico Prediction of ENST00000615487.1 Function

Given the predominantly nuclear localization of ENST00000615487.1 in normal colon epithelial cells and its cytoplasmic enrichment in CRC cell lines, further analyses were performed to investigate potential localization-dependent molecular interactions and functional roles of this transcript. Accordingly, interactions with genomic DNA were explored in the context of its nuclear localization, while potential interactions with miRNAs and proteins were investigated to assess cytoplasmic regulatory mechanisms in CRC cells.

#### 2.4.1. Prediction of Potential DNA Interactions of ENST00000615487.1

Potential interactions between ENST00000615487.1 and genomic DNA were analyzed using the Fasim-LongTarget tool. Genes with an integral interaction strength score greater than 500 were selected for further analysis. Among them, the strongest predicted interactions were identified for *HIP1R* (interaction score 1512) and *RPH3AL* genes (interaction score 1055) (Table 2).

**Figure 4:**
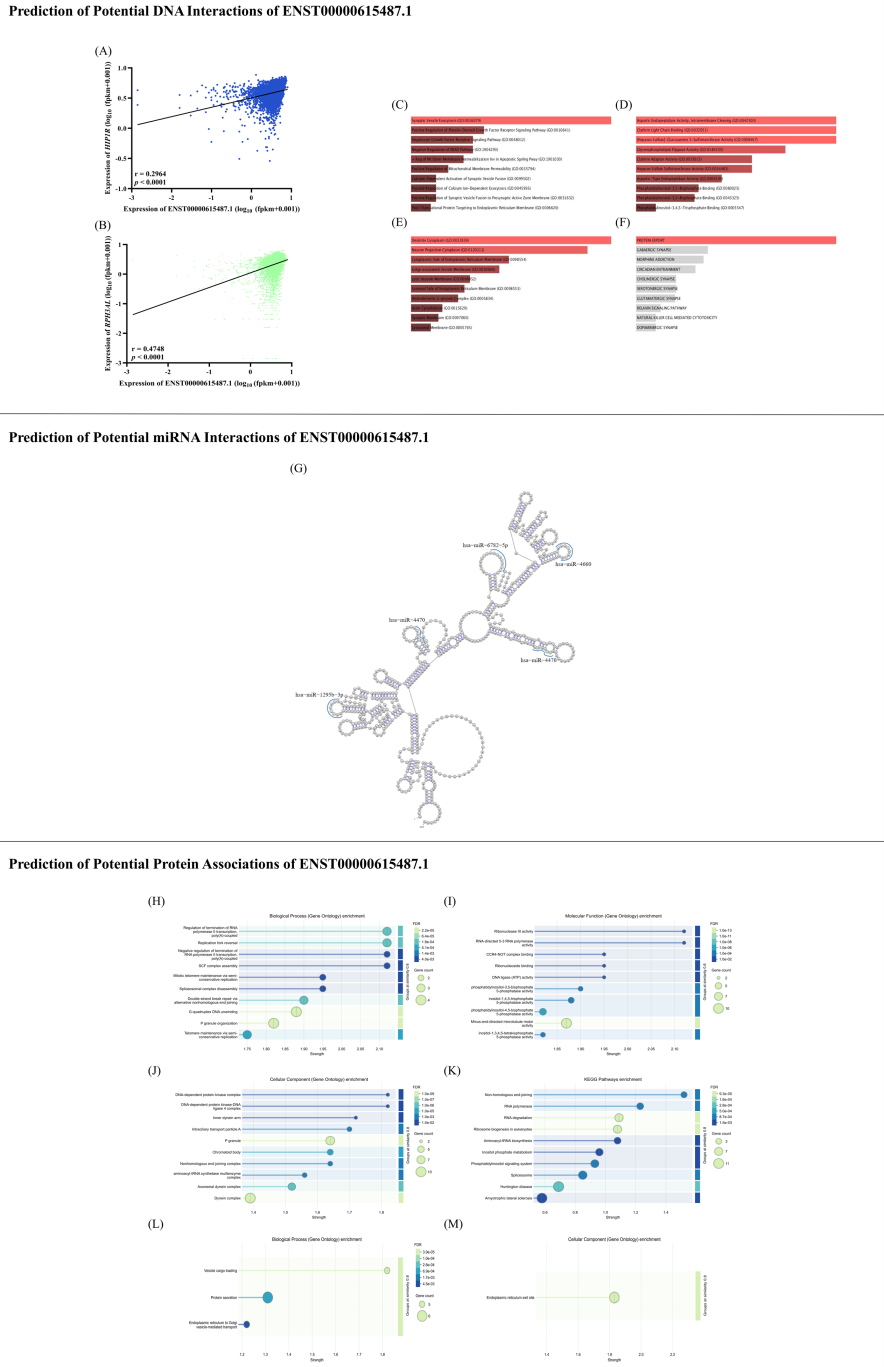
Functional characterization of ENST00000615487.1 through interaction with DNA, miRNAs and proteins coupled with enrichment analyses. (**A**,**B**) Correlation between ENST00000615487.1 expression and the expression of *HIP1R* (**A**) and *RPH3AL* (**B**) in CRC samples obtained from the UCSC Xena browser. (**C**–**F**) Functional enrichment analysis of genes predicted to interact with ENST00000615487.1 based on Fasim-LongTarget analysis and analyzed using Enrichr, showing enriched biological processes (**C**), molecular functions (**D**), cellular components (**E**), and KEGG pathways (**F**). (**G**) Secondary structure of ENST00000615487.1 generated using RNA Analyzer 3 showing predicted miRNA binding sites identified using the miRDB database. (**H**–**K**) Functional enrichment analysis of proteins predicted to interact with ENST00000615487.1 identified using AnnoLnc2 and analyzed using STRING, showing enriched biological processes (**H**), molecular functions (**I**), cellular components (**J**), and KEGG pathways (**K**). (**L**,**M**) Functional enrichment analysis of proteins positively correlated with ENST00000615487.1 expression in tumor tissue identified using AnnoLnc2 and analyzed using STRING, showing enriched biological processes (**L**) and cellular components (**M**). For enrichment analyses, the *x*-axis represents enrichment strength, circle color indicates false discovery rate (FDR)-adjusted significance, and circle size corresponds to the number of genes associated with each term.

To further evaluate the potential functional association between ENST00000615487.1 and the two genes with the highest predicted interaction scores, *HIP1R* and *RPH3AL*, expression correlation analysis was performed using CRC transcriptomic data obtained from the UCSC Xena browser. ENST00000615487.1 expression showed a statistically significant weak positive correlation with *HIP1R* expression (Spearman’s r = 0.2964, *p* < 0.0001) (Figure 4A) and a statistically significant moderate positive correlation with *RPH3AL* expression (Spearman’s r = 0.4748, *p* < 0.0001) (Figure 4B).

Afterward, the identified gene set was subjected to functional enrichment analysis using Enrichr to investigate associated biological processes, molecular functions, cellular components, and signaling pathways. Synaptic vesicle exocytosis emerged as the most significantly enriched biological process (Figure 4C). The most significantly enriched molecular functions included aspartyl endopeptidase activity, clathrin light chain binding, and heparan sulfate-glucosamine 3-sulfotransferase activity (Figure 4D). Cellular component analysis indicated enrichment in dendritic cytoplasm, neuronal projections, and membranes of the endoplasmic reticulum and Golgi apparatus (Figure 4E). KEGG pathway analysis highlighted protein export (Figure 4F).

#### 2.4.2. Prediction of Potential miRNA Interactions of ENST00000615487.1

Potential interactions between ENST00000615487.1 and miRNA molecules were analyzed using the miRDB database. Four miRNAs with predicted interaction scores greater than 70 were identified (Table 3). Among them, hsa-miR-4660 and hsa-miR-1295b-3p exhibited the highest interaction scores of 87 and 86, respectively. Predicted miRNA binding sites were mapped onto the secondary structure of ENST00000615487.1 generated using RNA Analyzer 3 (Figure 4G). Most predicted binding sites were localized within single-stranded loop regions of the transcript.

#### 2.4.3. Prediction of Potential Protein Associations of ENST00000615487.1

Potential protein interactors of ENST00000615487.1 were identified using the AnnoLnc2 database. A set of statistically significant candidate interacting proteins was retrieved (Table S2) and subsequently subjected to functional enrichment analysis using STRING to investigate associated biological processes, molecular functions, cellular components, and signaling pathways. STRING-based analysis indicated that these proteins are primarily involved in regulation of RNA polymerase II transcription termination, replication fork reversal, and SCF complex assembly (Figure 4H). Molecular function enrichment highlighted ribonuclease III activity, 5′–3′ RNA polymerase activity, CCR4-NOT complex binding, and ribonucleoside binding as the most significantly enriched terms (Figure 4I). Cellular component analysis revealed enrichment in the DNA-dependent protein kinase complex, DNA-dependent protein kinase–DNA ligase 4 complex, and the inner dynein arm (Figure 4J). KEGG pathway analysis further indicated involvement in non-homologous end joining, transcription, RNA degradation, and ribosome biogenesis pathways (Figure 4K).

Additionally, co-expression analysis of ENST00000615487.1 performed using the AnnoLnc2 database identified a set of proteins whose expression levels are positively correlated with ENST00000615487.1 expression in tumor tissue (Table S3). The identified protein set was further analyzed using STRING to investigate associated biological processes and cellular localization patterns. Functional enrichment analysis in STRING revealed significant overrepresentation of processes related to the cellular secretory pathway. The most significantly enriched biological processes included vesicle loading, protein secretion, and vesicular transport from the endoplasmic reticulum to the Golgi apparatus (Figure 4L). Consistently, cellular component analysis indicated enrichment at endoplasmic reticulum exit sites as the primary localization of these proteins (Figure 4M).

Furthermore, target genes (Table S4) regulated by the four miRNA molecules (Table 3) predicted to interact with ENST00000615487.1 were retrieved using the miRDB database. The obtained target gene set was then compared with the set of genes whose expression positively correlates with ENST00000615487.1 expression (Table S3). Two overlapping genes were identified between the two datasets. Specifically, hsa-miR-4660 was found to regulate the expression of *ZNF48*, while hsa-miR-1295b-3p targets *TMPRSS3*.

## 3. Discussion

Although recent studies have suggested a potentially important role of the ENST00000615487.1 transcript in carcinogenesis [8,9,10,11,12,13,14,15,16,17,18], its precise functional role in cellular processes remains insufficiently characterized. In addition, currently available data regarding the expression levels of ENST00000615487.1 in normal and tumor tissues are inconsistent across different malignancies [7,8,9,10,11,12,13,14,15,16,17,18]. Therefore, the present study aimed to further clarify the expression and biological role of this transcript, primarily in CRC, through a combination of in vitro and in silico analyses. We first examined the differential expression of ENST00000615487.1 across normal and tumor tissues of different cancer types, as well as in CRC cell lines. To further elucidate its potential biological role, we additionally investigated its cellular localization and experimentally validated its subcellular localization in CRC cells. Building upon these findings, subsequent analyses focused on the functional annotation of ENST00000615487.1 through the investigation of its potential interactions with DNA, miRNAs, and proteins.

Initial structural characterization performed using CPC2 and RNA Analyzer 3 demonstrated that ENST00000615487.1 lacks protein-coding potential, possesses a stable secondary structure, and exhibits characteristics consistent with long non-coding RNAs (lncRNAs), which is in agreement with its annotation in the Ensembl database. Previous studies have shown that structurally stable lncRNAs may play important roles in different stages of carcinogenesis [19]. In CRC, lncRNAs represent important regulators of signaling pathways involved in tumor initiation and progression through interactions with DNA, proteins, and miRNAs. Several well-characterized lncRNAs, including CCAT1, H19, lnc-ROR, PVT1, MALAT1, and HOTAIR, have been implicated in the regulation of proliferation, metastasis, stemness, chemoresistance, and Wnt/β-catenin signaling in CRC [5,6]. Collectively, these findings highlight the functional diversity of lncRNAs and their importance in colorectal tumorigenesis.

Previous studies have linked ENST00000615487.1 to multiple malignancies, including renal, ovarian, breast, endometrial, bladder, and gastric cancers [7,8,9,10,11,12,13,14,15,16,17,18]. Available data indicate that the biological role of this transcript may be highly tissue- and context-dependent. Although most studies suggest a protective or tumor-suppressive role, several reports indicate that its function may vary depending on the tumor type and cellular environment. For example, in kidney renal clear cell carcinoma (KIRC), ENST00000615487.1 has been identified among ferroptosis-related lncRNAs associated with improved prognosis and immune landscape regulation [9]. In ovarian cancer, it has been implicated in metabolic reprogramming and glycolysis regulation, whereas in breast cancer it was identified among the most dysregulated transcripts associated with multidrug resistance [8]. In bladder and gastric cancers, higher expression levels of ENST00000615487.1 have been associated with more favorable clinical outcomes [13,14].

Importantly, despite this growing body of evidence from bulk transcriptomic studies, the cellular source and cell type-specific expression patterns of ENST00000615487.1 remain largely unresolved. Previous analyses are primarily based on bulk RNA-sequencing datasets, where transcript abundance reflects an averaged signal across heterogeneous tumor, stromal, and immune compartments, and thus does not allow precise attribution of expression to specific cell populations. Moreover, no study has systematically investigated the single-cell-resolution distribution of ENST00000615487.1 across distinct cellular states within the colorectal tumor microenvironment. To address this limitation, we performed single-cell RNA sequencing analysis to determine the cellular specificity and transcriptional dynamics of ENST00000615487.1 across normal, adenoma, and carcinoma tissues, thereby providing a higher-resolution perspective on its expression landscape. The transcript was detected predominantly within epithelial cell populations, including enterocyte-like, goblet-like, mucin-secreting, invasive, stress-associated, and activated tumor epithelial states, while minimal expression was observed in immune and stromal compartments. Stem-like tumor cell populations were characterized by expression of WNT/stem-associated genes including ASCL2, AXIN2, RNF43, LGR5, OLFM4, and SLC12A2 [20], whereas invasive tumor epithelial cells exhibited elevated expression of MMP7, IGFBP2, SOX4, and TNFRSF12A [21,22,23]. Stress-associated tumor epithelial cells expressed genes such as S100P, LCN2, IFITM3, and NQO1 [24,25,26]. Although ENST00000615487.1 expression was generally detected in a relatively small subset of cells, carcinoma samples contained a higher proportion of transcript-positive cells compared to normal and adenoma tissues. However, this observation should be interpreted while considering the inherent technical limitations of droplet-based single-cell RNA sequencing platforms, including limited transcript capture efficiency and stochastic dropout events [27,28]. These limitations are particularly relevant for low-abundance transcripts, including many lncRNAs, which generally exhibit lower expression levels compared with protein-coding genes [29]. Therefore, the proportion of ENST00000615487.1-positive cells identified in this study may represent an underestimate of its actual distribution within the analyzed tissues. In contrast, transcript-positive normal epithelial cells exhibited higher mean expression intensity than tumor-associated populations. These findings suggest that ENST00000615487.1 expression undergoes dynamic remodeling during colorectal carcinogenesis, characterized by an expansion of transcript-positive epithelial populations accompanied by altered transcript abundance at the single-cell level. Furthermore, the predominant localization of ENST00000615487.1 within epithelial and tumor-associated cellular states supports its potential involvement in epithelial transformation, stress adaptation, and tumor progression.

Our analysis using the UCSC Xena browser further supports the tumor-specific nature of ENST00000615487.1 expression. Differential expression between normal and tumor tissues was observed in several malignancies, including elevated expression in THCA, CHOL, and KIRP, as well as reduced expression in COAD, UCEC, and KIRC. In contrast, no statistically significant differences were detected in PAAD, READ, or KICH samples. These findings support the hypothesis that the biological role of ENST00000615487.1 may depend on the specific tumor context and microenvironment.

PCR analysis performed in colon-derived cell lines detected ENST00000615487.1 expression in fibroblast (CCD-18Co) and CRC cell lines (HCT116, DLD1, and SW620), whereas expression was not detected in the non-tumorigenic epithelial cell line HCEC-1CT. These findings are consistent with our previously published NGS data obtained from spheroid cultures, which demonstrated elevated expression of ENST00000615487.1 in CRC cell lines (HCT116–12.01 FPKM, SW620–15.81 FPKM, DLD1–20.15 FPKM) but not in HCEC-1CT cells (0 FPKM) [7]. However, qRT-PCR analysis, which is more sensitive than conventional PCR, revealed detectable transcript levels in subcellular fractions of HCEC-1CT cells, although amplification occurred at very late cycles. Such late detection may indicate extremely low expression levels, non-specific amplification, or possible contamination.

Interestingly, while ENST00000615487.1 expression was not detected in intact HCEC-1CT cells by PCR, transcript expression was identified in certain normal colon tissue samples using the UCSC Xena browser. This discrepancy may reflect the presence of the transcript within stromal components of the tissue microenvironment. Supporting this hypothesis, our results additionally demonstrated the presence of ENST00000615487.1 in exosomes derived from HCEC-1CT, HCT116, and CCD-18Co cells, suggesting a potential role in intercellular communication within the tumor microenvironment. However, due to low RNA concentrations isolated from exosomes, equal input amounts could not be used for PCR analysis, preventing semiquantitative comparison between different cell lines. Exosomes are extracellular vesicles surrounded by a phospholipid bilayer and typically range from 50 to 100 nm in diameter. They contain various biomolecules, including DNA, mRNA, proteins, lipids, and non-coding RNAs such as lncRNAs [19]. Tumor-derived exosomes are known to contribute to cell signaling, immune modulation, tumor progression, and metastasis. In CRC specifically, exosomes have been implicated in multiple aspects of tumorigenesis and metastatic dissemination [19,30]. Nevertheless, the discrepancy between the expression pattern observed in primary tissues and cultured cell lines may also reflect inherent differences between in vivo and in vitro systems. In addition to the potential contribution of stromal cells within tissue samples, long-term cell culture may promote clonal selection, genetic drift, and transcriptional adaptation to artificial growth conditions, resulting in expression profiles that do not fully recapitulate those present in the native tissue microenvironment.

In silico localization analyses performed using lncATLAS, lncLocator, and iLoc-LncRNA (2.0) predicted predominantly cytoplasmic localization of ENST00000615487.1, which was consistent with the subcellular fractionation results obtained by qRT-PCR analysis in CRC cell lines. Analysis using lncATLAS demonstrated elevated cytoplasmic expression of the transcript in several cell lines, including GM12878, H1.hESC, HeLa.S3, HepG2, and MCF7, whereas nuclear enrichment was observed only in NHEK cells. Importantly, genes for cytoplasmic (*H19*) and nuclear (*MALAT1*) controls included in the lncATLAS analysis confirmed the reliability of the subcellular fractions investigated. In contrast to the CRC cell lines, subcellular fractionation followed by qRT-PCR suggested predominant nuclear localization of ENST00000615487.1 in the normal HCEC-1CT cell line. Because the subcellular localization of lncRNAs is closely associated with their molecular functions, the observed differences in the relative nuclear and cytoplasmic distribution of ENST00000615487.1 between HCEC-1CT and CRC cell lines may reflect a functional shift from transcriptional and chromatin-associated regulation toward post-transcriptional regulatory mechanisms in tumor cells. Accordingly, these observations motivated subsequent exploratory analyses investigating the potential functional role of ENST00000615487.1 through DNA interactions in normal cells and through miRNA and protein interactions in tumor cells, in accordance with its dominant localization pattern. Similar changes have previously been described in hematological malignancies, where altered subcellular distribution of lncRNAs alters their molecular interactions and biological functions [31].

Based on the assumption that nuclear localization in normal cells may facilitate interactions with genomic DNA and regulatory regions, potential DNA interactions of ENST00000615487.1 were analyzed using the Fasim-LongTarget tool. Sixteen genes displaying interaction scores higher than 500 were identified and subsequently analyzed using Enrichr. Functional enrichment analysis indicated that the protein products of these genes are involved in protein export and intracellular localization. Among the strongest predicted interactions were the *HIP1R* and *RPH3AL* genes. HIP1R participates in clathrin-mediated endocytosis [32,33,34,35] and has been implicated in the regulation of cell survival, receptor stability [36], and chromosome integrity through interactions with spindle microtubules [37]. Previous studies have demonstrated context-dependent functions of HIP1R in cancer biology, acting either as a tumor suppressor [32] or as a promoter of invasion depending on tumor type [38]. In CRC specifically, *HIP1R* has been proposed as a functional tumor suppressor involved in PD-L1 lysosomal degradation and immune checkpoint regulation [39,40]. Similarly, *RPH3AL* has been associated with vesicular transport and Ca^2+^-dependent exocytosis through interactions with Rab3A and cytoskeletal components [41]. Altered expression and mutations of *RPH3AL* have previously been linked to CRC tumorigenesis [41], although available data remain contradictory. While some studies reported reduced expression consistent with tumor-suppressive activity [41], others identified elevated protein levels and increased autoantibody production against RPH3AL in CRC patients [42]. The observed differential localization pattern of ENST00000615487.1 between normal and CRC cell lines may contribute to the deregulation of *HIP1R* and *RPH3AL* expression during malignant transformation. Supporting this hypothesis, correlation analysis using CRC tissue datasets from the UCSC Xena browser demonstrated weak and moderate positive correlations between ENST00000615487.1 expression and *HIP1R* and *RPH3AL*, respectively. These findings suggest a potential association between ENST00000615487.1 expression and the expression of *HIP1R* and *RPH3AL* in CRC; however, functional studies are required to determine whether a direct regulatory relationship exists.

Previous studies have emphasized the importance of lncRNA–miRNA interactions in CRC biology [5,6,43]. In contrast to its predominantly nuclear localization in normal cells, the predominantly cytoplasmic localization of ENST00000615487.1 in tumor cells suggests its potential involvement in post-transcriptional regulation through interactions with miRNAs. Using the miRDB platform, four miRNAs with interaction scores above 70 were identified: hsa-miR-4660, hsa-miR-1295b-3p, hsa-miR-4470, and hsa-miR-6782-5p. Secondary structure analysis demonstrated that the predicted binding sites of these miRNAs overlap with single-stranded loop regions of ENST00000615487.1, which are considered more accessible for regulatory interactions due to reduced structural stability compared to double-stranded regions. This observation may therefore support the likelihood of biologically relevant interactions. Further analysis of the regulatory roles of these miRNAs identified two overlapping target genes whose expression positively correlated with ENST00000615487.1 expression: *ZNF48* and *TMPRSS3*. *ZNF48* has previously been associated with oncogene-induced replication stress signatures in CRC [44], while *TMPRSS3* encodes a serine protease implicated in the progression and metastasis of several malignancies, including ovarian, breast, pancreatic, gastric, and nasopharyngeal cancers [45,46,47]. Numerous lncRNAs exert their regulatory functions through competitive binding of miRNAs, acting as molecular sponges that modulate signaling pathways involved in tumor progression [43]. In this context, ENST00000615487.1 may represent a candidate ceRNA interacting with hsa-miR-4660 and hsa-miR-1295b-3p. However, the proposed regulatory relationships involving ZNF48 and TMPRSS3 remain hypothetical and require experimental validation.

To further investigate the potential biological role of ENST00000615487.1, genes positively correlated with its expression in tumor tissue were identified using AnnoLnc2, followed by functional enrichment analysis using STRING. These analyses revealed enrichment of proteins involved in protein secretion, vesicle loading, and vesicular transport between the endoplasmic reticulum and Golgi apparatus. Notably, *DENND1C*, the antisense neighboring gene relative to ENST00000615487.1, was among the positively correlated genes. *DENND1C* has previously been implicated in endocytic recycling and membrane repair through interactions with Rab13 and Rab35 [48,49] and has been proposed to exhibit tumor-suppressive properties in lung adenocarcinoma [48]. Collectively, these findings suggest that ENST00000615487.1 may participate in the regulation of endocytosis, exocytosis, vesicular trafficking, and intracellular protein localization.

Potential biological functions of ENST00000615487.1 were additionally explored through the analysis of proteins predicted to interact directly with this transcript. Functional enrichment analysis demonstrated that these proteins are predominantly involved in transcription, replication, and DNA repair processes. These findings raise the possibility that ENST00000615487.1 may function as a molecular scaffold facilitating the recruitment of proteins involved in maintaining genomic stability and regulating the expression of tumor suppressor genes such as *HIP1R* and *RPH3AL*. Such a mechanism resembles previously described lncRNA-mediated scaffold functions, including the role of PiHL in regulating p53 stability in CRC, as well as the functions of LINP1, DDSR1, and NORAD in DNA damage repair and chromosomal stability maintenance [50].

Several limitations of this study should be acknowledged.

(1) A substantial part of the obtained results was based on in silico analyses and correlation-based approaches, which limits definitive conclusions regarding the direct molecular mechanisms of ENST00000615487.1. Importantly, the present study was not designed to establish causality or experimentally validate the predicted molecular functions of ENST00000615487.1. The proposed interactions with DNA, miRNAs, and proteins are based on computational predictions and correlation analyses and should therefore be considered hypothesis-generating. Functional experiments, including gain- and loss-of-function studies, RNA pull-down assays, RNA immunoprecipitation, and chromatin isolation approaches, will be necessary to determine whether the predicted interactions translate into biologically relevant mechanisms. Similarly, the classification of ENST00000615487.1 as a non-coding transcript was based on computational coding-potential prediction and database annotation and was not experimentally validated using approaches such as ribosome profiling, polysome association analysis, mass spectrometry, or in vitro translation assays.

(2) The study was conducted using a limited number of colorectal cell lines, which cannot fully recapitulate the complexity and heterogeneity of patient tumors and the tumor microenvironment.

(3) An additional limitation concerns the experimental assessment of subcellular localization. Although nuclear and cytoplasmic fractionation coupled with qRT-PCR supported differential localization of ENST00000615487.1 between HCEC-1CT and CRC cell lines, the predominant cytoplasmic localization observed in CRC cells was consistent with computational predictions. However, the purity of the isolated fractions was evaluated only using the cytoplasmic marker *GAPDH*. A nuclear reference transcript was not included, limiting the assessment of fraction purity and the exclusion of potential cross-contamination between fractions. Furthermore, imaging-based validation methods such as RNA fluorescence in situ hybridization (RNA-FISH) or confocal microscopy were not performed. Therefore, the observed localization patterns should be interpreted with caution and require further validation in future studies.

(4) ENST00000615487.1 transcript detection in exosomes was based on conventional PCR from exosomal cDNA, which confirms presence but does not allow quantitative assessment of transcript abundance. In addition, while exosomes were enriched using a validated commercial column-based isolation kit reported to yield intact vesicles in the expected exosomal size range, and whose underlying isolation chemistry has been independently validated for canonical exosomal markers (CD63, CD81, TSG101) [51], we did not perform morphological characterization (e.g., TEM, NTA) or immunoblotting for canonical exosomal markers (CD63, CD81, TSG101) in the present study, and therefore cannot formally confirm that the isolated fraction represents a pure, well-defined exosome population as recommended by current MISEV2018 reporting guidelines [52]. Future studies incorporating EV marker validation, morphological characterization, and quantitative PCR-based methods will be necessary to confirm the exosomal origin of ENST00000615487.1 and to determine its relative abundance and functional relevance.

(5) Finally, while correlation analyses suggested functional associations between ENST00000615487.1 and genes such as *HIP1R* and *RPH3AL*, further mechanistic and functional studies are necessary to determine whether these relationships are causal and to clarify the biological role of this transcript in colorectal carcinogenesis.

Overall, the results of the present study provide an initial characterization of ENST00000615487.1 expression patterns, cellular and subcellular localization, and predicted molecular associations. The findings generate testable hypotheses regarding potential localization-dependent functions of this transcript that warrant further mechanistic investigation.

## 4. Materials and Methods

### 4.1. Cell Line Cultivation

In this study, a panel of adherent human colon cell lines representing different stages of CRC progression, as well as normal epithelial and stromal components of the colon tissue, was used. The panel comprised a non-tumorigenic colonic epithelial cell line (HCEC-1CT), primary colon adenocarcinoma cell lines (HCT116 and DLD1), a metastatic adenocarcinoma cell line (SW620) and a colonic fibroblast cell line (CCD-18Co). All procedures involving cell lines were performed under sterile conditions. Cells were maintained in an incubator with an automated supply of 5% CO_2_ at a temperature of 37 °C. The growth medium used was DMEM (Dulbecco’s Modified Eagle’s Medium) (1×) + GlutaMAX^TM^-I (Waltham, MA, USA), supplemented with 4.5 g/L D-glucose and pyruvate (Gibco, Thermo Fisher Scientific, Waltham, MA, USA), and further enriched with 10% FBS (Fetal Bovine Serum, Sigma Aldrich, St. Louis, MO, USA) and 1% Antibiotic-Antimycotic (Gibco, Thermo Fisher Scientific, Waltham, MA, USA). Subcultivation was performed every 3–4 days when the culture reached approximately 80–90% confluence. After removing the culture medium, the cells were washed once with a 1×PBS (Phosphate-Buffered Saline) solution, followed by the addition of a 1× trypsin-EDTA solution (0.5 mg/mL trypsin and 0.2 mg/mL EDTA; Sigma-Aldrich, St. Louis, MO, USA). Cells were then incubated at 37 °C for 3–5 min. Trypsin was inactivated by adding a complete medium at a volume three times greater than that of the trypsin used. The cells were subsequently resuspended and seeded into appropriate culture vessels.

### 4.2. Establishment of a Cell Model Overexpressing the ENST00000615487.1 Transcript

The complementary DNA (cDNA) sequence of the ENST00000615487.1 transcript was cloned into the pcDNA3.1 vector, named here the pcDNA3.1/ENST00000615487.1 construct. The HCEC-1CT cell line was transiently transfected using the FuGENE HD reagent (Promega, Madison, WI, USA) in order to overexpress the ENST00000615487.1 transcript, and transfection efficiency was confirmed via quantitative real-time polymerase chain reaction (qRT-PCR). HCEC-1CT cells with transient ENST00000615487.1 overexpression were utilized as positive controls in polymerase chain reaction (PCR) analyses.

### 4.3. RNA Isolation

#### 4.3.1. Total RNA Isolation, Genomic DNA Removal, and RNA Quantification

Total RNA isolation from cell pellets was performed using 1 mL of TRI Reagent^TM^ Solution (Invitrogen, Thermo Fisher Scientific, Waltham, MA, USA) according to the manufacturer’s instructions. Isolated RNA was stored at −80 °C.

To eliminate traces of genomic DNA, RNA isolates were treated with Deoxyribonuclease I, Amplification Grade (Invitrogen, Waltham, MA, USA). Digestion was carried out using 1 U (International Unit) of enzyme per μg of RNA, with the addition of the corresponding reaction buffer in an equal volume. Following a 15 min (min) incubation at room temperature, the reaction was terminated by adding 25 mM EDTA and heating at 65 °C for 10 min. After DNaseI treatment, RNA concentration and purity were measured again using the BioSpec-nano device (Shimadzu Corp., Kyoto, Japan).

The concentration and purity of the isolated RNA were determined by measuring absorbance at 260 nm and 280 nm using a BioSpec-nano spectrophotometer (Shimadzu Corp., Japan).

The same RNA quality assessment and DNase treatment procedures were applied to RNA isolated from subcellular fractions and exosomes prior to downstream analyses.

#### 4.3.2. RNA Isolation from Subcellular Fractions

The separation and RNA isolation from nuclear and cytoplasmic subcellular fractions from cells cultured in T-75 flasks were carried out using the commercial Cytoplasmic & Nuclear RNA Purification Kit (Norgen Biotek Corp., Thorold, ON, Canada) according to the manufacturer’s instructions. Isolated RNA was stored at −80 °C until further use.

#### 4.3.3. RNA Isolation from Exosomes

For the isolation of RNA from exosomes derived from the culture medium of cells grown in two T-75 flasks, the commercial Cell Culture Media Exosome Purification Kit (Norgen Biotek, Thorold, ON, Canada) was used following the manufacturer’s protocol. Isolated RNA was stored at −80 °C until further use.

### 4.4. Complementary DNA (cDNA) Synthesis

The High-Capacity cDNA Reverse Transcription Kit (Applied Biosystems, Foster City, CA, USA) was used for cDNA synthesis according to the manufacturer’s protocol, using a Biometra TProfessional TRIO Thermocycler (Analytic Jena, Jena, Germany). In total, 1 μg of total RNA was converted to cDNA.

### 4.5. Polymerase Chain Reaction (PCR)

To detect the ENST00000615487.1 transcript in the cultivated cell lines, a polymerase chain reaction (PCR) was conducted using the DNA Polymerase kit (EURx, Gdańsk, Poland). Primers for the ENST00000615487.1 transcript were designed using Primer3 [53,54,55]. The sequences of the primers were: ENST00000615487.1 F: 5′-AGGCAGTCATCCCCAAAGAA-3′, ENST00000615487.1 R: 5′-CCCACGGCCCCTTTAAAATT-3′, and the product length was 229 bp. The PCR amplification was performed using a Biometra TProfessional TRIO Thermocycler with the following thermal profile: 95 °C for 5 min, followed by 30 cycles of (95 °C for 30 s, 60 °C for 30 s, 72 °C for 30 s), and a final extension at 72 °C for 10 min. The success and specificity of the PCR were verified by 2% agarose gel electrophoresis. A 100 bp DNA Ladder (Invitrogen, Thermo Fisher Scientific, Waltham, MA, USA) was used to confirm the relative size of the PCR products. Visualization was performed using the BioDocAnalyse system equipped with a CCD camera (Biometra, Jena, Germany).

### 4.6. Quantitative Real-Time Polymerase Chain Reaction (qRT-PCR)

The relative expression levels of the ENST00000615487.1 transcript and *GAPDH* gene were analyzed via qRT-PCR using PowerUp SYBR Green chemistry (Applied Biosystems, Foster City, CA, USA). The primers used for ENST00000615487.1 were identical to those previously described for PCR, while the specific primers for *GAPDH* were as follows: forward, 5′-GTGAAGGTCGGAGTCAACG-3′ and reverse, 5′-TGAGGTCAATGAAGGGGTC-3′, with a 229 bp product length (designed using Primer3). Amplification was performed using a Line Gene 9600 Plus Fluorescent Quantitative Detection System (BIOER Technology, Hangzhou, China) with the following thermal profile: 95 °C for 10 min, followed by 40 cycles of (95 °C for 15 s, 60 °C for 1 min). Data were collected and processed using the Power Gene 9600 Plus software (FQD-96a V1.0.13, BIOER Technology, Hangzhou, China). To confirm reaction specificity, a melting curve analysis was performed after each run. Each sample was analyzed in triplicate.

For subcellular localization analysis, equal amounts of DNase-treated RNA from the nuclear and cytoplasmic fractions were used for cDNA synthesis and subsequent qRT-PCR. Because the aim of this analysis was to compare the relative distribution of the same transcript between cellular compartments rather than quantify differential gene expression, no housekeeping gene was used for normalization. Instead, the relative nuclear and cytoplasmic distribution of ENST00000615487.1 was calculated directly from Ct values using the ΔCt approach, while *GAPDH* was analyzed separately as a cytoplasmic fractionation control. The results of the qRT-PCR subcellular localization analysis were expressed using the following formula:

$${{log}_{2}2}^{\left(C_{t\;nucleus}-C_{t\;cytoplasm}\right)}$$

### 4.7. In Silico and Transcriptomic Analysis

The nucleotide sequence of the ENST00000615487.1 transcript was retrieved in FASTA format from the Ensembl genome browser using the GRCh38 version of the human genome (https://www.ensembl.org/index.html, accessed on 24 November 2025). The general characteristics of the ENST00000615487.1 transcript were analyzed using the Coding Potential Calculator 2 [56,57] (CPC2) and RNA Analyzer 3 [58] tools. Specifically, CPC2 utilizes an alignment-free logistic regression model based on four intrinsic sequence features (peptide length, amino acid composition, Fickett score, and open reading frame score) to robustly differentiate non-coding from coding transcripts. Concurrently, RNA Analyzer 3 was employed as an integrated platform to screen for regulatory and structural features; it utilizes the Rfam database for regulatory motif identification and incorporates the RNAfold algorithm from the ViennaRNA package to predict RNA secondary structure, structural folding characteristics, and highly structured regulatory regions.

To predict the subcellular localization of the ENST00000615487.1 transcript, the lncLocator [59] and iLoc-lncRNA 2.0 [60] tools were utilized. These complementary predictive tools were employed to leverage distinct machine learning frameworks and sequence-feature extraction methods to improve consensus reliability; lncLocator applies a stacked ensemble strategy combining support vector machine and random forest classifiers trained on both k-mer frequencies and deep learning-generated sequence abstractions, whereas iLoc-lncRNA 2.0 utilizes a support vector machine (SVM) architecture operating on key octonucleotide (8-tuple) features integrated into a general Pseudo K-tuple Nucleotide Composition framework.

Additionally, the lncATLAS bioinformatics database [61] was employed. Data within the lncATLAS database are based on the GENCODE release V24 annotation. The relative concentration of the gene across different cellular compartments was quantified using the Cytoplasmic/Nuclear Relative Concentration Index (CN RCI), calculated according to the following formula:

$$CN\;RCI\;=\;\left(\frac{{Fraction}_{i\;}\left(RPKMs\right)}{{Fraction}_{j}\left(RPKMs\right)}\right)\;$$

where *Fraction_i_* represents the cytoplasmic fraction, *Fraction_j_* represents the nuclear fraction expressed through RPKM (Reads Per Kilobase of transcript per Million mapped reads).

To predict the cellular function of the ENST00000615487.1 transcript, the degree of its interaction with proteins, DNA molecules, and other RNA molecules was analyzed. For this purpose, the following machine learning-based bioinformatics tools were used: (1) Fasim-LongTarget [62,63,64]—a bioinformatics tool that predicts genome-wide interactions between long non-coding RNA molecules and DNA sequences by employing an optimized local alignment algorithm based on 24 canonical Hoogsteen and reverse Hoogsteen triplex-forming rules; (2) miRDB [65,66]—a database used to investigate potential interactions between the target transcript and miRNA molecules via the MirTarget algorithm, which utilizes a machine learning framework trained on high-throughput sequencing data to evaluate common binding and target downregulation features; (3) AnnoLnc 2 [67,68]—a bioinformatics tool used to analyze the interaction of the ENST00000615487.1 transcript with proteins and its co-expression with other genes (co-expression networks in cancer samples are derived using Pearson correlation coefficients filtered through a step-down threshold algorithm (*r* from 0.9 to 0.7) to minimize false positives, while RNA-protein interactions are determined via a dual approach combining experimental CLIP-Seq peak-calling (utilizing Piranha and PARalyzer algorithms on data from the POSTAR2 database) and ab initio prediction using the lncPro tool evaluated against an empirical NULL distribution (*p* < 0.01)); (4) STRING [69]—a functional protein association network database utilized to map physical and functional interactions based on integrated data from experimental repositories, text-mining, and computational predictions; and (5) Enrichr [70,71,72]—a comprehensive gene set enrichment analysis tool used to statistically evaluate the overrepresentation of candidate genes across diverse biological pathways and Gene Ontology terms using Fisher’s exact test.

The expression analysis of the ENST00000615487.1 transcript in several tumor and non-tumor tissues, as well as the expression of the *HIP1R* and *RPH3AL* genes in CRC only, was performed using the UCSC Xena Browser [73] platform. The TCGA Pan-Cancer cohort was selected for analysis, and expression data were downloaded from the TOIL RSEM FPKM dataset. To analyze expression profiles across different types of malignancies, samples were classified according to phenotypic parameters: sample type (to differentiate between primary tumor, metastasis, and healthy tissue) and primary disease (to identify specific histopathological tumor types). To provide a focused overview of malignancies with the highest transcript abundance, tumor types were ranked according to the mean expression level of ENST00000615487.1 across all available samples, and the ten tumor types with the highest expression were selected for further differential expression analysis. The numbers of tumor and normal samples included for each cancer type are provided in Table S1.

Single-cell RNA sequencing data were obtained from the GEO dataset GSE161277. Raw 10x Genomics counts matrices were imported and merged into a single Seurat object. Quality control filtering excluded cells with low gene counts, high gene counts, or high mitochondrial transcript content. Cell clustering was performed using Seurat graph-based clustering following construction of a shared nearest neighbor (SNN) graph based on the first 15 principal components. Clusters were identified using the Louvain algorithm with a resolution parameter of 0.5. Cell type annotation was performed manually based on CellMarker 2.0 [74], PanglaoDB [75] and previously reported cell lineage markers from the literature. Expression of the ENST00000615487.1 transcript (annotated as ACO10503.4 in this dataset) was further evaluated across different cellular populations and tissue conditions. All analysis was performed in R (version 4.5.1) using the Seurat [76,77,78,79,80] package.

### 4.8. Statistical Data Analysis

Differential expression of the ENST00000615487.1 transcript between tumor and normal tissues obtained from the UCSC Xena Browser was evaluated using Welch’s t-test. Because multiple comparisons were performed across the ten selected tumor types, *p*-values were adjusted using the two-stage step-up false discovery rate (FDR) procedure of Benjamini, Krieger, and Yekutieli, with a desired FDR (Q) of 5%. Adjusted q-values < 0.05 were considered statistically significant.

The correlation between the expression of the ENST00000615487.1 transcript and the *HIP1R* and *RPH3AL* genes was assessed using Spearman’s correlation. For single-cell RNA sequencing analyses, comparisons between different tissue groups were performed using the Wilcoxon rank-sum test. Across all analyses, differences and correlations were considered statistically significant at *p* < 0.05. GraphPad Prism software, version 10.6.1 (Dotmatics, Boston, MA, USA), was used for statistical analyses and data visualization.

## 5. Conclusions

The findings of this study identify ENST00000615487.1 as a potentially relevant lncRNA associated with CRC biology. Coding potential analysis supported the current annotation of ENST00000615487.1 as a non-coding transcript, while expression analyses revealed a deregulated expression pattern across different malignancies, including reduced CRC expression. Single-cell transcriptomic analysis further demonstrated that ENST00000615487.1 expression is predominantly restricted to epithelial cell populations, particularly enterocyte-like, goblet-like, mucin-secreting, and tumor-associated epithelial states, with minimal expression in immune and stromal compartments. Moreover, carcinoma samples exhibited a higher proportion of ENST00000615487.1-positive cells, suggesting altered transcript regulation during colorectal carcinogenesis. Furthermore, the detection of this transcript in exosomal fractions indicates that the transcript may be released into the extracellular environment and warrants further investigation regarding its potential role in intercellular communication and modulation of the tumor microenvironment.

Both computational prediction and subcellular fractionation-based analyses suggested differences in the relative subcellular distribution of ENST00000615487.1, with predominant nuclear localization observed in HCEC-1CT cells and predominant cytoplasmic localization observed in CRC cell lines. These findings suggest that ENST00000615487.1 localization may differ between normal and malignant cellular contexts and may be associated with context-dependent functions. However, further validation using imaging-based approaches will be required to confirm these localization patterns and determine their functional relevance during colorectal carcinogenesis.

Computational analyses identified candidate DNA-, protein-, and miRNA-associated networks potentially linked to ENST00000615487.1. These analyses highlighted genes and pathways related to vesicular trafficking, protein localization, transcriptional regulation, DNA replication, DNA repair, and post-transcriptional regulation. However, these findings are based on bioinformatic predictions and correlation analyses and therefore should be considered exploratory and hypothesis-generating rather than evidence of direct molecular mechanisms.

Overall, the present study provides a comprehensive characterization of the expression profile, cellular and subcellular distribution, and predicted molecular associations of ENST00000615487.1. These findings establish a descriptive and hypothesis-generating framework for future functional studies aimed at determining the mechanistic role of this lncRNA in CRC and evaluating its potential clinical relevance.

## Figures and Tables

**Table 1:** Structural characteristics and coding potential analysis of ENST00000615487.1 using CPC2 and RNA Analyzer 3.

Ensembl ID	Length (bp)	Free Energy (kcal/mol)-RNA Analyzer 3	Number of Loops-RNA Analyzer 3	Number of Hairpins-RNA Analyzer 3	Coding Potential (Probability)-CPC2 and RNA Analyser 3
ENST00000615487.1	688	−204.8	27	14	non-coding transcript (0.02)

**Table 2:** Genes predicted to interact with ENST00000615487.1 with an integral interaction strength score > 500 based on Fasim-LongTarget analysis.

Ensembl ID	Gene Name	Interaction Strength
**ENSG00000130787**	***HIP1R***	**1512**
**ENSG00000181031**	***RPH3AL***	**1055**
ENSG00000237940	*AC093642.3*	764
ENSG00000167414	*GNG8*	724
ENSG00000005206	*SPPL2B*	626
ENSG00000103227	*LMF1*	618
ENSG00000068650	*ATP11A*	615
ENSG00000256484	*RP13-507P19.1*	615
ENSG00000162040	*HS3ST6*	576
ENSG00000250632	*RP3-513G18.2*	566
ENSG00000223729	*RP11-145E17.2*	563
ENSG00000087266	*SH3BP2*	534
ENSG00000176945	*MUC20*	530
ENSG00000104969	*SGTA*	526
ENSG00000115155	*OTOF*	508
ENSG00000005206	*SPPL2B*	506

**Table 3:** Predicted interactions between ENST00000615487.1 and miRNA molecules identified using miRDB.

miRNA Name	miRNA Sequence	Interaction Score	Seed Region Binding Position
hsa-miR-4660	UGCAGCUCUGGUGGAAAAUGGAG	87	470
hsa-miR-1295b-3p	AAUAGGCCACGGAUCUGGGCAA	86	174
hsa-miR-4470	UGGCAAACGUGGAAGCCGAGA	80	268, 558
hsa-miR-6782-5p	UAGGGGUGGGGGAAUUCAGGGGUGU	73	338

## Data Availability

Single-cell RNA sequencing data analyzed in this study were obtained from the publicly available Gene Expression Omnibus (GEO) dataset GSE161277. Differential expression of ENST00000615487.1 across 10 tumor types was analyzed using the publicly available UCSC Xena Browser platform. Experimental datasets generated during this study are available from the corresponding author upon reasonable request.

## Supplementary Materials

The following supporting information can be downloaded at: https://www.mdpi.com/article/10.3390/ncrna12040024/s1, Table S1: Full Names, Abbreviations, and Number of Tumor and Paired Normal Samples; Table S2: Statistically significant proteins predicted to interact with ENST00000615487.1 identified using AnnoLnc2; Table S3: Proteins positively correlated with ENST00000615487.1 expression in tumor tissue identified using AnnoLnc2; Table S4: Predicted target genes of miRNAs interacting with ENST00000615487.1 using miRDB.

## Abbreviations

The following abbreviations are used in this manuscript:

bp	base pairs
cDNA	complementary DNA
ceRNA	competing endogenous RNA
CN RCI	Cytoplasm-to-Nucleus Relative Concentration Index
CPC2	Coding Potential Calculator 2
CRC	Colorectal cancer
DMEM	Dulbecco’s Modified Eagle’s Medium
FBS	Fetal Bovine Serum
FDR	False Discovery Rate
KIRC	Kidney Renal Clear Cell Carcinoma
lncRNA	Long non-coding RNA
min	Minute
miRNA	microRNA
NGS	Next-Generation Sequencing
NHEK	Normal Human Epidermal Keratinocyte
PBS	Phosphate-Buffered Saline
PCR	Polymerase Chain Reaction
qRT-PCR	Quantitative Real-Time Polymerase Chain Reaction
RNA-seq	RNA sequencing
RNA-FISH	RNA Fluorescence In Situ Hybridization
RPKM	Reads Per Kilobase of transcript per Million mapped reads
SNN	Shared Nearest Neighbor graph
U	International Unit
